# Epigenetics, human imprintome, and chronic diseases

**DOI:** 10.1042/EBC20253015

**Published:** 2025-07-04

**Authors:** Randy L. Jirtle

**Affiliations:** 1 Department of Biological Sciences, North Carolina State University, Raleigh, NC, U.S.A; 2 Center for Human Health and the Environment, North Carolina State University, Raleigh, NC, U.S.A; 3 Toxicology Program, North Carolina State University, Raleigh, NC, U.S.A

**Keywords:** chronic diseases, DOHaD, epigenetics, genomic imprinting, imprintome, radiation hormesis

## Abstract

Two epigenetically labile subsets of genes that link embryonic environmental exposures with adult disease susceptibility are those that are imprinted and those with metastable epialleles. The expression of genes with metastable epialleles, like the agouti gene in Agouti viable yellow (Avy) mice, is highly variable between individuals but uniform in tissues within an individual. We used the Avy mouse to demonstrate that exposure to nutritional supplements, chemical toxicants, and low-dose ionizing radiation during embryogenesis alters adult disease susceptibility by modifying the epigenome. Genomic imprinting is a unique species-dependent epigenetic form of gene regulation that evolved approximately 150 million years ago in a common ancestor to Therian mammals. It resulted in monoallelic parent-of-origin-dependent gene silencing. Thus, imprinted genes are functionally haploid disease susceptibility loci, since only a single genetic or epigenetic event is required to alter their function. Expression of imprinted genes in the human genome is regulated by hemi-methylated imprint control regions (ICRs) in the human imprintome. Furthermore, human imprintome ICRs associated with chronic diseases (e.g., cancer, diabetes, and obesity) and behavioral disorders (e.g., autism, bipolar disorder, psychopathy, and schizophrenia) can now be identified with the use of cells from peripheral samples and the human imprintome array. The importance of metastable epialleles and imprinted genes in the etiology of environmentally induced human chronic diseases is discussed in this review.

## Introduction

Epigenetics was first defined by the English developmental biologist Conrad Waddington [1] as ‘…the interactions of genes with their environment which bring the phenotype into being’. Holliday and Pugh [2] proposed in 1975 that covalent chemical DNA modifications, including methylation of cytosine at CpG dinucleotide sites, were the molecular mechanisms behind Conrad’s hypothesis. Subsequently, epigenetics was defined as the study of changes in gene expression that occur not by changing the DNA sequence but by modifying DNA methylation and remodeling [3]. Only 100 papers were published on epigenetics in 1990, but the number increased to 17,000 in 2024. This 170-fold increase in annual publications in 34 years demonstrates that the field of epigenetics is growing exponentially with a doubling time of four years.

### Developmental origin of health and disease

Forty years ago, Barker and Osmond demonstrated that mortality rates from chronic diseases in England and Wales in the 1970s were correlated geographically with increased death rates among newborn babies caused by poor nutrition [4]. These findings resulted in the novel postulate that retardation of growth during critical periods of development in fetal life, and subsequent low birth weight, were associated in adulthood with chronic diseases including cancer, cardiovascular disease, and bronchitis [5]. Subsequent epidemiologic studies of survivors of the Dutch famine during World War II (1944–1945) and the Chinese Great Leap Forward famine (1959–1961) provided additional evidence that significant reduction in food availability, particularly during the first trimester of pregnancy, increases the adult risk of developing a variety of chronic diseases such as cancer, cardiovascular disease, diabetes, kidney disease, obesity, and schizophrenia [6-8]. Moreover, there is evidence that the increased risk of these chronic diseases can be inherited transgenerationally, possibly by changes in the epigenome [8-10].

These results ultimately evolved into the developmental origins of health and disease (DOHaD) hypothesis that posits the intriguing idea that the evolution of developmental plasticity, which enables an organism to adapt to environmental signals during early life, can also increase the risk of developing chronic diseases when there is a mismatch between the perceived environment during gestation and that which is actually encountered in adulthood [11]. This postulate, however, was met with skepticism because at the time, there was no known mechanism linking molecular changes induced by environmental exposures during early development to disease formation decades later.

With the use of the Agouti viable yellow (A^vy^) mouse, we demonstrated in 2003 that exposure to enhanced methyl donor supplements during embryogenesis induced persistent DNA methylation changes at the *Agouti* locus that resulted in alterations in adult phenotype [12]. Our study provided a plausible mechanism for DOHaD and provided the first evidence that epigenetic modifications (e.g., DNA methylation) in early life impacts later health [13]. These findings ushered in the era of environmental epigenomics [14], and the first international meeting on this subject was held in 2005; it can be viewed at https://www.geneimprint.com/site/meetings/2005-durham.

There is now evidence that two epigenetically labile targets—imprinted genes and those with metastable epialleles—link environmental exposures during early embryonic development to adult diseases. Their potential role in the etiology of environmentally induced chronic diseases and the strategies to investigate their influence on human health and disease are discussed in this review.

## Metastable epialleles

### Agouti mouse study—nutritional supplements

The highly variable expression of genes with metastable epialleles results from stochastic allelic changes in the epigenome (e.g., DNA methylation) during early embryonic development rather than from mutations in the genome. Genes with metastable epialleles have large variability in expression between individuals but low variability in gene expression between tissues in an individual. Thus, the ratio of these two gene-expression variances is expected to be large for genes with metastable epialleles.

The formation of the embryonic DNA methylation that regulates metastable epiallele expression is controlled by both the level of methyl donors in the diet (e.g., folic acid, vitamin B_12_, choline chloride and betaine), and the environmental conditions that modify the efficiency of one-carbon metabolism. Additionally, the activity of DNA methyltransferase 3 alpha (DNMT3A) and DNA methyltransferase 3 beta (DNMT3B) functions in *de novo* methylation [15], and DNA methyltransferase 1 (DNMT1) functions in maintenance DNA methylation [16]. Thus, any chemical or physical exposure during early development that affects either methyl donor levels or DNMT3A, DNMT3B, and/or DNMT1 activity can modify the DNA methylation of metastable epialleles, thereby altering their expression in adulthood and the pathogenesis of disease formation.

The most actively investigated metastable genes in mice are the *agouti* gene in the A^vy^ mouse [17], the *Axin^Fu^* gene [18], and the *Cabp^IAP^* gene [19], although additional metastable epialleles have been identified in the mouse [20]. Moreover, metastable epialleles or correlated regions of systemic interindividual variation (CoRSIVs) have been identified in cattle [21] and the human [22-24], indicating that these systemic epigenetic variants are common in mammals.

**Figure 1:**
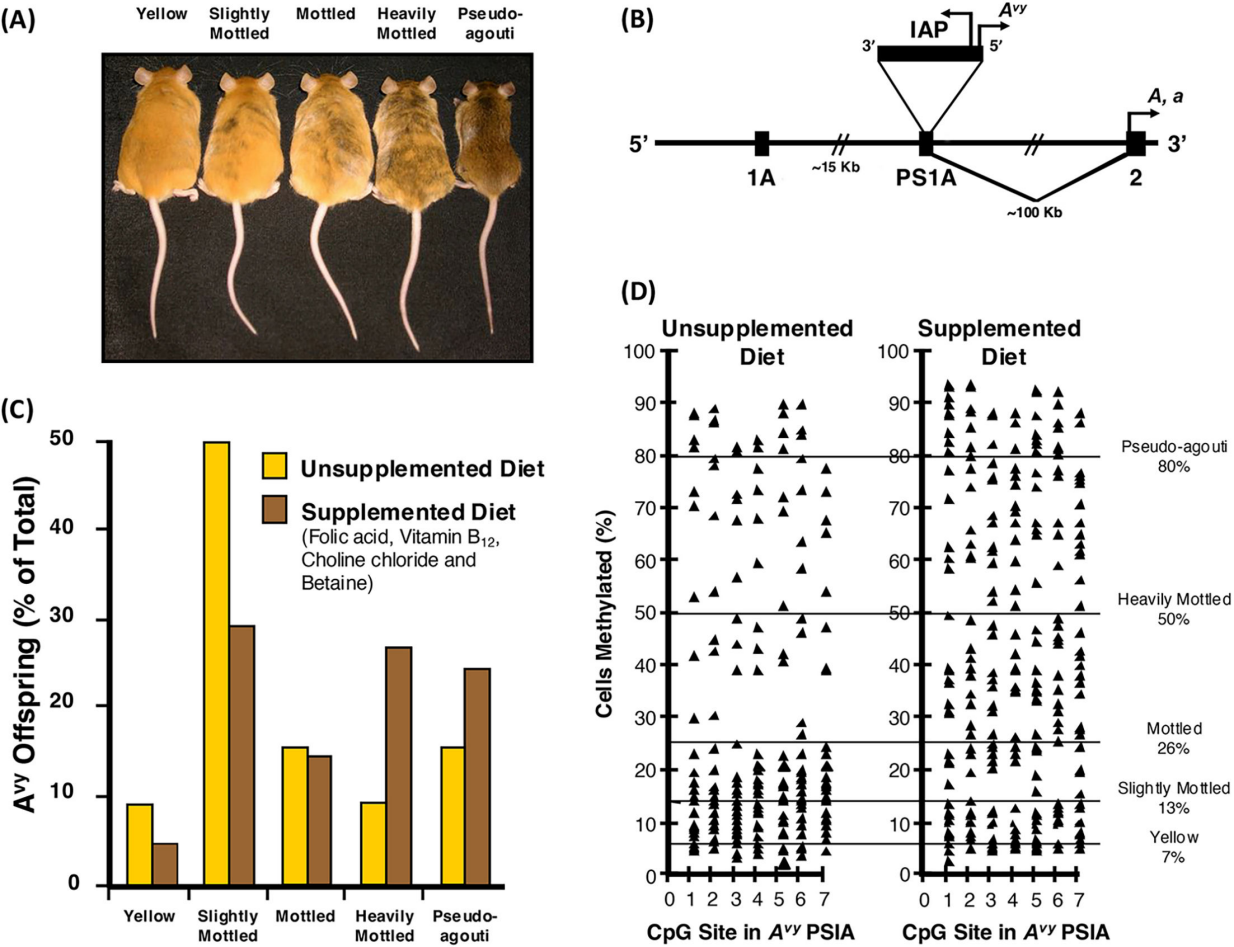
Agouti mouse nutritional supplement study. (**A**) Isogenic A^vy^/a mouse littermates representing the five coat-color phenotypes. (**B**) The contraoriented IAP insertion within PS1A of the murine *Agouti* gene. A cryptic promoter (i.e., short arrow labeled *A^vy^*) drives ectopic *Agouti* expression. CpG sites 1–7 are oriented in the 3´ to 5´ direction with respect to the IAP insertion. Transcription of A and a alleles initiates from a hair-cycle-specific promoter in exon 2 (i.e., short arrow labeled A,a). (**C**) Coat color distribution of *A^vy^/a* offspring born to mothers fed an unsupplemented diet (*i.e*. yellow bars) and a methyl donor supplemented diet (*i.e*. brown bars). (**D**) CpG methylation within the *A^vy^* PS1A of *A^vy^/a* offspring from mothers fed unsupplemented and methylation supplemented diets; redrawn from [12,25].

The A^vy^ mouse varies in coat color, ranging from brown (i.e., methylated intracisternal A-particle [IAP]) to yellow (i.e., unmethylated IAP) with intermediate mottled A^vy^ mice that are epigenetically mosaic because it harbors a metastable *Agouti* gene (Figure 1A). A retroviral IAP is inserted approximately 100 kb upstream of the gene (Figure 1B) [26,27]. The degree of methylation of CpG sites in the IAP at the cryptic promoter region in the proximal end of the IAP varies dramatically among individual isogenic A^vy^ mice, causing a wide distribution in coat color. Hypomethylation of this alternative promoter results in inappropriate *Agouti* gene expression throughout the A^vy^ mouse. This not only leads to a yellow coat color; it also antagonizes MC4R in the hypothalamus, causing the animals to overeat, thereby becoming obese, and subsequently developing diabetes and cancer at a high frequency. In contrast, the incidence of these diseases is markedly reduced in pseudoagouti (i.e., brown) offspring that develop when this ectopic promoter is hypermethylated, and the developmental expression of the *Agouti* gene is limited to the hair follicles [28-30].

This makes the A^vy^ mouse an excellent, exquisitely sensitive biosensor for identifying environmental exposures, early in development, that alter adult disease susceptibility by modifying the epigenome rather than by mutating the genome. When the A^vy^ mice were exposed during pregnancy to nutritional donor supplements (i.e., folic acid, vitamin B_12_, choline chloride and betaine), the coat color of their offspring shifted to heavily mottled and brown (Figure 1C) concomitant with a significant increase in DNA methylation of IAP at the A^vy^ locus (Figure 1D) [12]. A plausible mechanism for DOHaD had been identified (***ShortCutstv*** Documentary: *The Agouti Mouse Study* - https://www.youtube.com/watch?v=VM37fh5ykbg).

Genistein is a major phytoestrogen in soy linked to cancer chemoprevention and decreased adipose deposition. Maternal A^vy^ mouse dietary genistein supplementation during gestation, to levels comparable with humans consuming high-soy diets, also shifted the coat color of the A^vy^/a offspring toward brown [25]. This marked phenotypic change was likewise associated with increased methylation of the IAP upstream of the transcription start site of the *Agouti* gene (Figure 1B). The extent of this DNA methylation was similar in tissues from the three germ layers, indicating that genistein acts during early embryonic development. Moreover, this genistein-induced hypermethylation persisted into adulthood, decreasing ectopic *agouti* expression and protecting the offspring from obesity. Genistein is not a methyl donor, and no evidence was found of enhanced efficiency of one or more steps in the one-carbon metabolism pathway. Thus, the effect of genistein on IAP methylation during early embryonic development may be mediated by modifying the activity of DNMT3A, DNMT3B [15] and/or DNMT1 [16]. This was the first evidence that *in utero* dietary genistein affects gene expression and alters susceptibility to obesity in adulthood by permanently altering the epigenome.

### Agouti mouse study—nongenotoxic agents

Perinatal exposure to the endocrine active compound, bisphenol A (BPA), a chemical used in the manufacture of polycarbonate plastic, epoxy resin, and other polymer materials, is associated with higher body weight, increased breast and prostate cancer, and altered reproductive function [31]. Maternal exposure of A^vy^ mice to this endocrine disruptive, non-genotoxic compound [32] shifted the coat color distribution of the mouse offspring toward yellow by decreasing DNA methylation at the IAP of the *Avy* locus (Figure 1B); DNA methylation was also decreased at the *Cabp^IAP^* metastable epiallele locus [33]. DNA methylation at the *A^vy^* locus was similar in tissues from the three germ layers, providing evidence that epigenetic patterning during early stem cell development is sensitive to BPA exposure. Moreover, maternal dietary supplementation, with either methyl donors or the phytoestrogen genistein, blocks the negative DNA hypomethylating effect of BPA. These results provide compelling evidence that early developmental exposure of A^vy^ mice to BPA increases offspring disease susceptibility by stably altering the epigenome, an effect that can be counteracted by maternal dietary supplements. Thus, food is medicine!

### Agouti mouse study—low-dose ionizing radiation

The negative human health effects resulting from moderate to high-dose exposures (i.e., > 100 cGy) have been well documented since the discovery of ionizing radiation in 1895; however, the majority of human exposures occur in the low-dose range (i.e., < 10 cGy). In addition to inducing genetic mutations, low-dose ionizing radiation (LDIR) may also alter the epigenome.

We first demonstrated over a decade ago that LDIR exposure of the A^vy^ mouse during pregnancy causes positive adaptive changes in the offspring by significantly increasing DNA methylation at the *Agouti* locus (Figure 1B) [34]. Average DNA methylation was significantly increased in offspring exposed to doses between 0.7 and 7.6 cGy, with maximum effects at 3 cGy; this effect was seen in both male and female mice, but was significantly greater in males. Offspring coat color was concomitantly shifted toward brown, which is linked to reduced obesity, diabetes, and cancer in the A^vy^ mouse. Thus, LDIR exposure of A^vy^ mice *in utero* results in the offspring being healthier than those that are not exposed to radiation. The phenomenon that occurs when an environmental factor that is toxic at high doses stimulates a beneficial adaptive response in an organism at low doses is called hormesis [35].

Interestingly, maternal dietary antioxidant supplementation negated the LDIR-induced coat color shift of the offspring to brown, the increased IAP DNA methylation, and the positive adaptive health responses in the offspring exposed to 3 cGy [34]. Consequently, LDIR exposure during gestation elicits epigenetic alterations that lead to positive adaptive phenotypic changes that are mitigated with antioxidants. This indicates the hormetic effects are mediated in part by oxidative stress. These findings provide evidence that in the isogenic A^vy^ mouse, epigenetic alterations resulting from LDIR play a role in radiation hormesis; however, they do not define the repertoire of genes whose expressions are altered by exposure to LDIR. This would require the use of next-generation sequencing technologies, like those developed by Oxford Nanopore Technologies (Oxford, UK) [36], to determine the human methylome in a radiation dose-dependent manner. This long-read sequencing technology can directly determine the DNA methylation of CpG sites, including regions that are difficult to map using short-read methods, making it particularly useful for studying differential methylation throughout the human genome [37].

**Figure 2:**
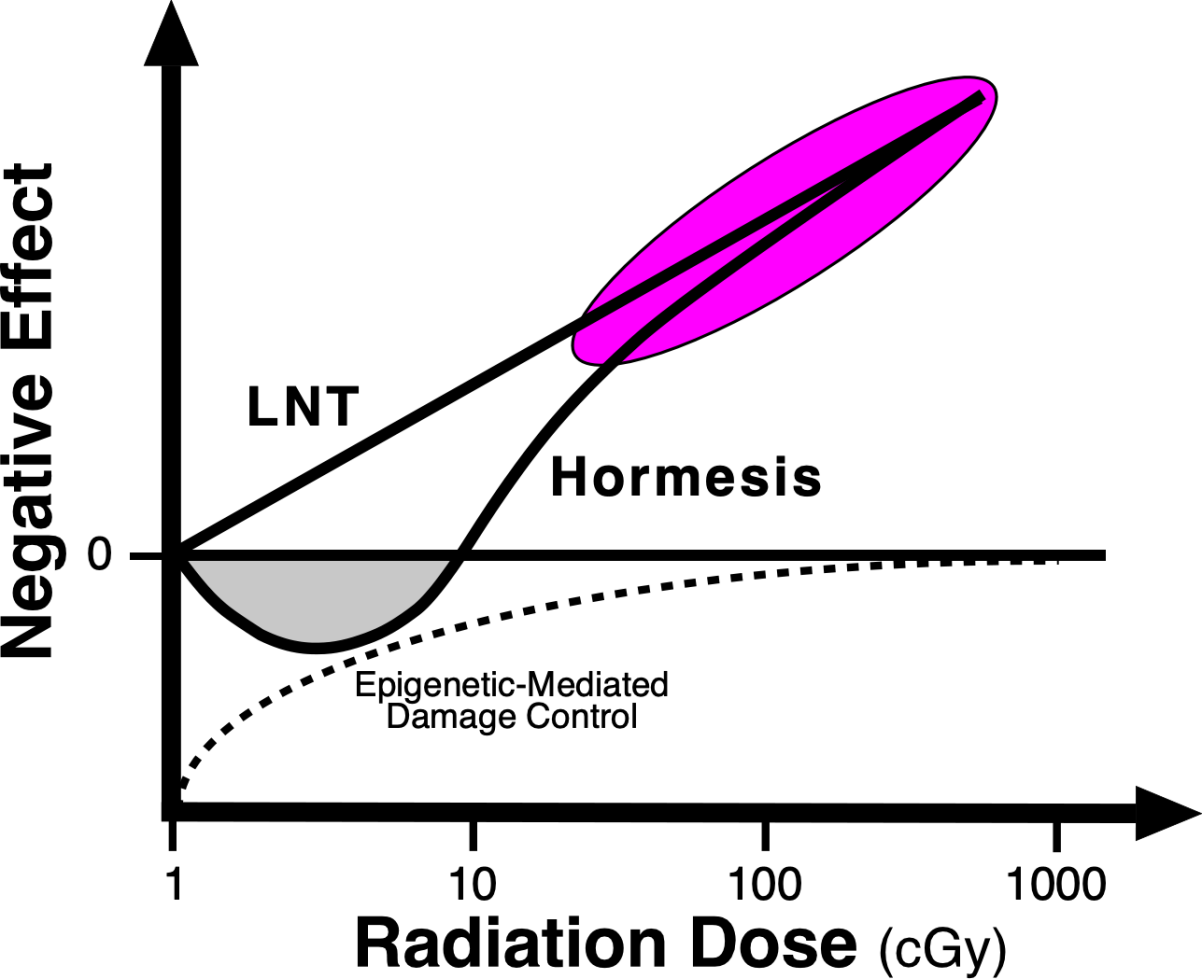
LNT and hormesis radiation dose response models. Linear no-treshold (LNT) and hormesis radiation risk assessment models of negative biological effect (e.g., unhealthy yellow *A^vy^* mice, cancer incidence) versus radiation dose overlap at high doses where most radiation response data are collected (magenta ellipse) but vary markedly in their assessment of risk in the hormetic region at doses < 10 cGy (gray area). Exposure of *A^vy^* mice *in utero* to LDIR results in a decrease in the incidence of unhealthy yellow *A^vy^* offspring with a concomitant increase in the healthy brown offspring. The nadir of this epigenetically regulated hormetic effect is at 1–3 cGy [34]; redrawn from [38].

Our findings bring into question the assumption that every dose of radiation is harmful, as required by the LNT radiation risk model (Figure 2). Although our important epigenetic LDIR study is not often discussed in the radiation risk literature, it makes a critical contribution to the debate that occurred over a quarter of a century ago on the importance of radiation hormesis in human risk assessment and health.

At the turn of the millennium, the argument concerning the scientific validity of radiation hormesis was quite contentious and remains so today. This conflict ultimately resulted in a debate in 1998 between John Cameron, Professor of Medical Physics at the University of Wisconsin, and John Moulder, Professor of Radiation Oncology at the Medical College of Wisconsin, on whether radiation hormesis occurs at low doses of radiation exposure [38]. Cameron presented a significant amount of experimental animal and human epidemiological data in support of radiation hormesis, which ultimately resulted in his publishing a review article on this subject where he concluded, ‘We need increased background radiation to improve our health’ [39]. Moulder argued against the phenomenon of hormesis, principally because *no known* biological mechanism existed to support its existence.

Our LDIR A^vy^ mouse study is not only consistent with the existence of radiation hormesis but also provides direct evidence that the mechanism of radiation hormesis involves epigenetic modifications that alter the regulation of gene expression [34]. If ionizing radiation only caused genetic mutations, the LNT model shown in Figure 2 would be correct; however, LDIR also modifies the epigenome—the cellular programs that tell the genes when, where, and how to work. At low radiation doses, this gives rise to the radiation dose-dependent positive adaptive changes observed in our A^vy^ LDIR study that maximize at 3 cGy and are lost near 10 cGy (Figure 2). This dose response is described best by a radiation hormesis model of radiation risk assessment, *not* the LNT model.

Hormesis is a biphasic dose-response phenomenon where low doses of radiation induce positive adaptive effects, while high doses lead to harmful effects [40]. Hormetic dose response relationships are present at the cell, organ, and individual level and involve bystander communications. The bystander effect also results from changes in the epigenome, rather than mutations in the genome, since it is blocked by the elimination of the DNA cytosine methyltransferases, DNMT1 and DNMT3A [15].

The role of hormesis in determining cancer risk at low doses of radiation has become of particular importance, since the increasing number of people receiving CT scans raises the possibility of increased radiation-induced malignancies in the population based upon the LNT model of radiation risk assessment [41-43]. Cancer risk could also potentially be increased from high natural background radiation [44] and nuclear power plant disasters [45]. A recent study demonstrated, however, that a preponderance of studies, using higher quality case control and cohort methodology, indicate that cumulative radiation doses up to 100 mSv do not increase cancer risk, consistent with the findings in a number of other experimental and human studies [39,44,46]. Because of the paucity of cancer response data at doses < 10 cGy and its high variability, the ability of epidemiological studies to discriminate between the LNT and hormetic dose response models is low [40].

Thus, rather than continuing to debate about the existence of radiation hormesis [38], the time has come to use next-generation DNA methylation sequencing techniques [36] to define the repertoire of gene regulatory regions and the genes and biochemical pathways they control that are altered epigenetically in a radiation dose-dependent manner. It will be interesting to see if LDIR is beneficial to human health, as postulated over two decades ago by Cameron [38], because it significantly alters epigenetically the expression of genes involved in 1) damage prevention by temporarily stimulating detoxification of molecular radical species; 2) damage repair by temporarily stimulating repair mechanisms; 3) damage removal by stimulating apoptosis; and 4) damage removal by stimulating the immune response. We presently need this information to accurately define human risk to LDIR and to identify novel ways to protect humans, as we become more reliant on nuclear power for electricity and venture deeper into space.

## Imprinted genes

Genomic imprinting is an example of intergenerational epigenetic inheritance where the transmission of parental genomes to the mammalian embryo results in epigenetically mediated, parentally biased gene expression. Thus, it results in an exception to Mendel’s Laws of inheritance.

**Figure 3:**
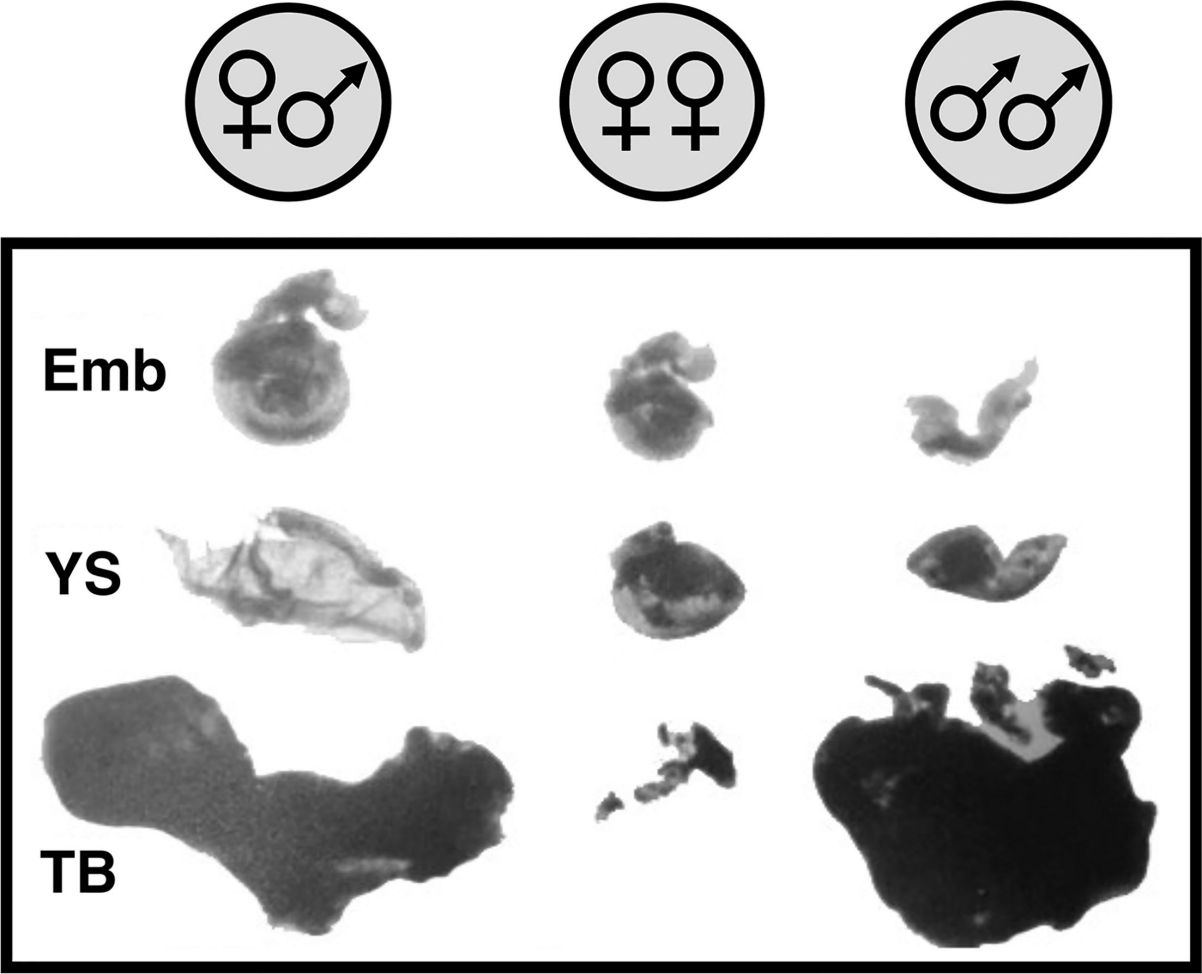
Maternal and paternal genomes are not functionally equivalent. Normal mouse development occurs when one pronucleus is female and the other is male. Mouse nuclear transplantation studies show abnormal development of the trophoblasts and embryo in diploid gynogenotes derived from two female pronuclei and diploid androgenotes derived from two male pronuclei, respectively; redrawn from [47,48].

The first experimental evidence that the parental genomes of mammals are not functionally equivalent came from elegant mouse nuclear transplantation studies in the mid-1980s (Figure 3) [47-49]. They demonstrated that the abortive pregnancies resulting from diploid gynogenotes derived from two female pronuclei are markedly different from those that developed from diploid androgenotes derived from two male pronuclei. The former displayed severe placental defects, while the latter had marked embryonic growth retardation. Thus, in contrast to parthenogenesis being observed in ﬁsh, amphibians, reptiles, and birds, it does not normally occur in Therian mammals [50,51]. Interestingly, however, it has now been shown that of all the imprint control regions (ICRs) in the mouse genome, the paternally methylated germline ICRs regulating imprinting at the *Igf2/H19* and *Dlk1/Dios* imprinted domains are the only paternal barriers for the development of parthenogenesis in mice [52].

In 1974, the *T-maternal effect* (*Tme*) locus was reported with parent-of-origin effects on the viability of mice with small deletions in chromosome 17 [53]. Using this murine model, Denise Barlow identified in 1991 the first imprinted gene, *Igf2r,* which is expressed only from the maternal allele [54]. In addition to the scavenging of Igf2, Igf2r is involved in the intracellular trafficking of lysosomal enzymes, TGFβ1 activation, T cell-mediated apoptosis, and the internalization of extracellular phosphomannosyl glycoproteins [55]. Additionally, the Igf2r plays a central role in mediating the signaling pathways required for Igf2 to facilitate memory and synaptic plasticity [56]. Soon after *Igf2r* was identified to be imprinted, *Igf2* was demonstrated to be imprinted and paternally expressed [57]. The following year, the first human gene identified to be imprinted was the maternally expressed *H19* [58]. This gene was subsequently found to form a reciprocally imprinted domain at chromosome location 11p15.5 with paternally expressed *IGF2* [59]. The era of genomic imprinting research had begun, and there are now approximately 143 genes experimentally demonstrated to be imprinted in mice, 120 in humans, and 23 in marsupials (https://www.geneimprint.com/site/genes-by-species).

### Evolution of genomic imprinting

Some imprinted genes in eutherian mammals (*e.g*. human and mouse) are likewise imprinted in marsupials (e.g., wallaby and opossum) while others are uniquely imprinted only in Eutherians or marsupials [60-63] with the first *NNAT* being the first Eutherian-specific imprinted gene identified [64]. In contrast, genes tested for imprint status in the monotremes (e.g., echidna and platypus) and Aves (e.g., chicken) are biallelically expressed [65-68]. These findings are consistent with genomic imprinting originating in the Jurassic period approximately 150 MYA in a common ancestor to marsupials and eutherians [65,67,69,70]. The phenomenon of genomic imprinting also evolved independently in some insects [71] and flowering plants [72]. The functional haploidy resulting from a gene being imprinted enables a single genetic mutation or epigenetic modification to alter the function of an imprinted gene, making imprinted genes unique disease susceptibility loci.

Scientists have struggled to formulate a theory to explain the adaptive evolutionary advantage of imprinted genes, since their development eliminates the protection that diploidy affords against the deleterious effects of recessive mutations. A number of theories have been proposed to explain its evolution, such as the co-adaption theory, the genome defense theory, and the conflict/kinship theory (see reviews [60,73,74]). A comparison of the paternal expression of the growth factor, *Igf2* [57], and the maternal expression of *Igf2r* [54], the receptor involved in the degradation of *Igf2*, highlights their opposite effects on growth. These opposite effects led to the formulation of the most prominent and actively debated theory for the evolution of genomic imprinting, the parental kinship or conflict hypothesis [75,76]. This theory maintains that genomic imprinting developed in response to viviparity and polygamy and speculates that fitness effects during placental development were the principal factors that shaped its evolution. For the first time in evolutionary history, the placenta exists as an interface in which both the paternal and maternal genomes can exert their influence on resource allocation within the intrauterine environment. Postnatal nurturing behavior, like lactation, further extends this interaction between the mother and offspring, indicating that the mammary gland may be the functional equivalent of the placenta in the postnatal stage of eutherian mammal development [77].

Consequently, imprinted genes are not only prominently expressed in the placenta and mammary gland but also half of known imprinted genes are imprinted in the brain and affect behavior [62,78]. Pioneering mouse chimeric studies demonstrated that gynogenote/WT chimeric embryos developed abnormally large brains, whereas androgenote/WT chimeras had small brains [79]. Furthermore, the gynogenote cells were not distributed randomly throughout the brain, but rather were located in the cortex, striatum, and hippocampus; whereas, the androgenote cells were enriched in the hypothalamus. These results provide evidence that genomic imprinting may have facilitated a rapid expansion of the mammalian brain and altered behavioral development over evolutionary time. These findings are also consistent with the imprinted brain theory, an extension of the conflict theory, which posits that skewed paternal and maternal expression of imprinted genes results in the behavioral conditions of autism and schizophrenia, respectively [80,81]. The identification of the human imprintome [82] and the imprintome array [83], coupled with the development of high-throughput DNA sequencers, may finally allow for this novel postulate to be experimentally tested.

According to the conflict theory, genes expressed from the paternal allele favor increased maternal investment to enhance an offspring’s own fitness at a cost to all others. In contrast, genes expressed from the maternal allele maximize reproductive fitness of the mother, ensuring availability of resources for all of her current and future progeny. This postulate explains successfully the parental allelic expression bias of a number of imprinted genes, including the paternal expression of *Igf2, a* growth enhancer, and the maternal expression of *Igf2r, a* growth inhibitor [75,84]. It likewise is consistent with the opposing growth effects of *Dlk1* and *Grb10* [85]. Supporting evidence for this postulate also comes from the discovery of imprinted genes in eutherian mammals and marsupials, but not in the egg-laying monotremes or birds [62,65,67,68,70], another prediction of the conflict theory [75].

### Imprinting mechanisms

Genomic imprinting appears to have evolved independently at least three times. In the mealybug, imprinting is manifested as heterochromatization of the entire paternal genome selectively in males [86]. In *Arabidopsis*, genes are imprinted primarily in the nutrient-providing endosperm, and a number have been identified to be imprinted, including *MEA* and *PHE1,* which are maternally and paternally expressed, respectively [87].

**Figure 4:**
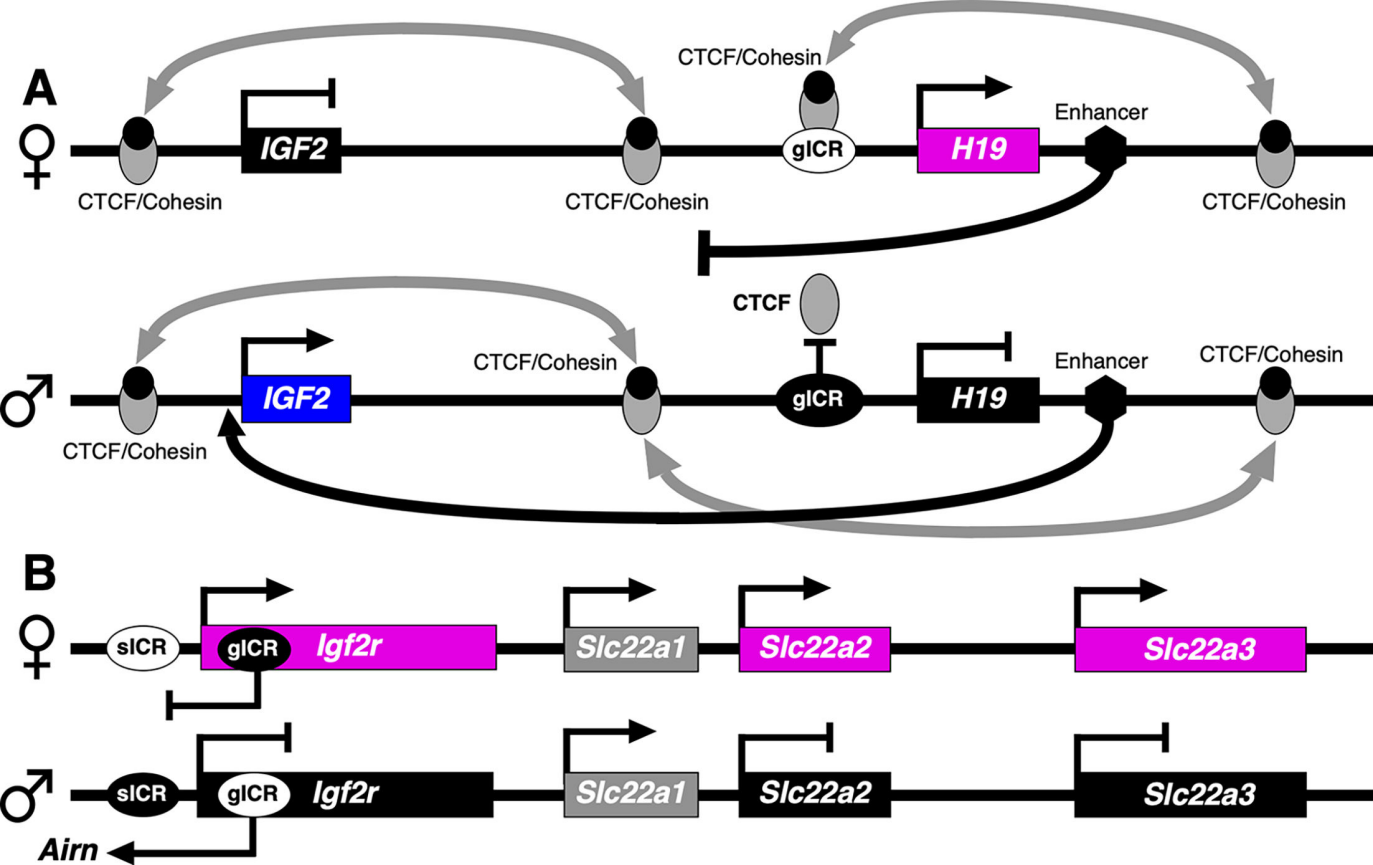
Models of imprinted gene expression control in mammals. (**A**) Chromatin boundary imprinting model, and (**B**) noncoding RNA imprinting model; redrawn from [90]. Black box, unexpressed gene, black circle, cohesin, blue box, paternally expressed gene, black oval, methylated ICR; gray box, biallelically expressed gene; light gray oval, CTCF; magenta box, maternally expressed gene, white oval, unmethylated ICR. ICR, imprint control region.

**Figure 5:**
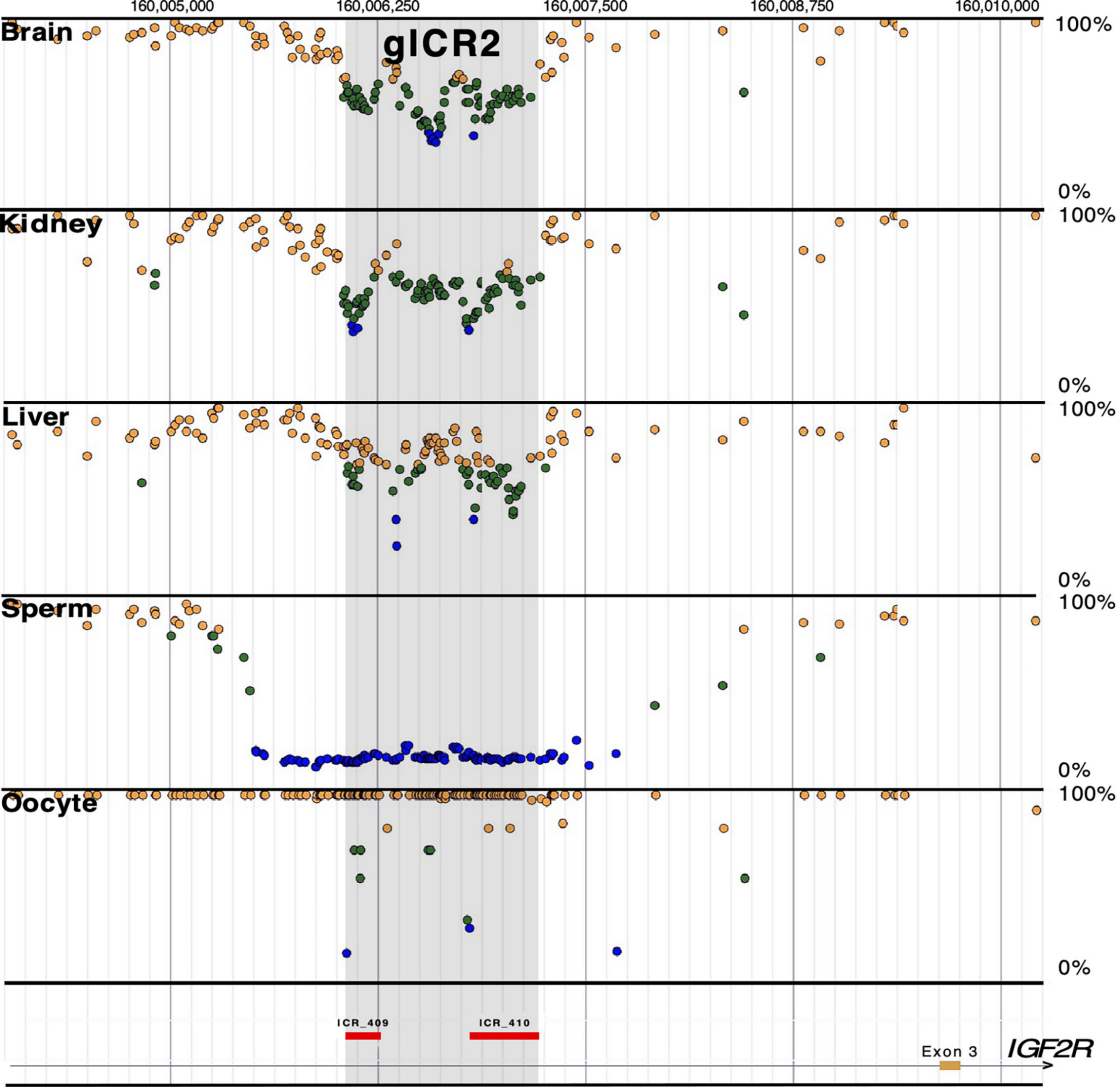
Candidate ICR for human *IGF2R*. WGBS identified a candidate gICR (gray rectangle) in intron 2 of the human *IGF2R* (ICR_409 and ICR_410, red rectangles) in tissues from the three embryonic germ layers (i.e., brain, kidney, and liver); the average percent methylation in these tissues is approximately 50%. The average DNA methylation in sperm and oocytes is 0% and 100%, respectively. Dots indicate hemi-methylated (green), hypomethylated (blue) and hypermethylated (yellow) CpG sites [82], https://humanicr.org/. gICR, germline imprint control regionICR, imprint control region; WGBS, whole genome bisulfite sequencing.

Both classical or canonical and non-canonical genomic imprinting exist in eutherian mammals [88,89]. Canonical imprinting is regulated by ICRs that are differently methylated at CpG sites in a parental-dependent manner (Figure 4 and Figure 5) [82,90]. The ICRs can be hundreds to thousands of bases in length [82], and since imprinted genes are frequently clustered, control one or more proximal genes (Figure 4). Canonical imprinted genes participate in metabolism, embryonic and placental growth, and brain development. In contrast, non-canonical imprinted genes are not regulated by differentially methylated ICRs, as in canonical imprinting. This form of genomic imprinting relies on histone modifications like H3K27me3. It primarily occurs in the maternal germline and affects gene expression in the developing embryo, particularly in the placenta [88,89].

Canonical imprinting mechanism in vertebrates is more complex than in insects and plants. The inherited gametic imprint regulatory marks necessarily undergo a cycle involving their establishment in the PGCs of one generation, maintenance during somatic cell divisions throughout life in the resulting individual, and erasure and re-establishment in the germ cells during embryogenesis to reflect the sex of the individual in which they reside [14,91,92]. DNA methylation is a candidate for this inherited marking system, since it can be modulated with the help of the *de novo* methyltransferases DNMT3A, DNMT3B, and DNMT3L in PGCs and maintained throughout life with the aid of DNMT1 [93-95]. Proteins like CTCF, coupled with its testis-specific counterpart CTCFL, non-coding RNAs, and methyl-CpG-binding domain proteins, which recruit histone deacetylases, are all involved in imprinting regulation, emphasizing the complexity of the imprinting process [92,96,97].

The ICRs that regulate canonical genomic imprinting are present in imprinted gene clusters where discrete *cis*-acting DNA elements that carry a heritable epigenetic mark distinguish the two parental alleles [92]. A germline imprint control region (gICR) in Eutherians contains DNA methylation at specific CpG sites, resulting in both a methylated and unmethylated allele in somatic cells. For these gICRs, the inherited methylation marks can be paternally derived from the sperm, as in the gICR located upstream of *H19* in the *IGF2/H19* cluster (Figure 4A) [92,98] or maternal in origin like the gICR in intron 2 of murine *Igf2r* (Figure 4B).

The ICRs have to be protected from the wave of DNA demethylation that occurs soon after fertilization, or the parental imprint memory of the ICRs would be lost. DPPA3 protects imprinted genes in the zygote from active demethylation by inhibiting 5mC conversion to 5hmC [99]. In rodents, *Zfp57* and *Zfp445* cooperate in preserving the ICRs during cleavage divisions [100]. In humans, *ZFP445* appears to play a larger role in this process [100-102]; however, mutations in *ZFP57* are present in individuals with transient neonatal diabetes who show a variable pattern of DNA hypomethylation at imprinted loci throughout the genome [103]. Interestingly, we predict *ZFP57* (ICR_383) to potentially be imprinted while *ZFP445* is not predicted to be imprinted [82].

CpG-rich sequences can also acquire gICR-dependent, parental-specific DNA methylation somatic imprint control region (sICR) marks around the time of implantation. An example is the sICR in the promoter region of mouse *Igf2r* that is methylated on the paternal allele (Figure 4B) [104,105]. Additionally, since the gICR imprint regulatory marks may not always be ‘read’, monoallelic expression of imprinted genes can be dependent upon cell type, developmental stage, and sex of the individual, resulting in significant cell type variation in monoallelic expression [106,107]. It has been proposed that this epigenetically mediated variation in imprinted gene expression may have played a role in mammalian speciation [108]. This would help explain why the repertoires of imprinted genes vary among Therian mammals [109,110] (https://www.geneimprint.com/site/genes-by-species).

The parent-of-origin-dependent expression of imprinted genes is mediated by at least two different imprinting mechanisms. Briefly, the chromatin boundary imprinting model holds that allele-specific modifications at ICRs affect binding of insulator proteins (*e.g*. CTCF), thereby mediating gene silencing. Such is the case for the *IGF2/H19* imprinted domain (Figure 4A) [111]. On the paternal allele, CTCF does not bind the methylated gICR, resulting in distinct maternal/paternal patterns of cohesin-mediated chromatin remodeling around *H19*. This results in maternal expression of *H19* and the silencing of *IGF2* isolated from the downstream enhancer. In contrast, the CTCF/cohesin interactions on the paternal allele bring the downstream enhancer into range of the IGF2 promoter, activating its expression [90].

In the noncoding RNA imprinting model, the production of mouse *Airn* is critical for the establishment of imprinting (Figure 4B) [92]. During early development of the embryo and placenta, paternal allele silencing only requires transcriptional interference of the *Airn* transcript originating from the unmethylated gICR and overlapping of the *Igf2r* promoter; however, in late development, the paternal allele is methylated at the sICR [112]. The paternal alleles of two additional genes, *Slc22a2* and *Slc22a3,* are also paternally silenced, but in a more tissue-dependent manner than *Igf2r*. DNA methylation on the maternal allele of the gICR inhibits the production of *Airn* from the mother’s copy of the gene. Thus, the sICR on the maternal allele remains unmethylated, permitting expression of *Igf2r, Slc22a2,* and *Slc22a3* from only the maternal allele. The adjacent gene, *Slc22a1,* escapes imprinting control and is biallelically expressed.

As in the mouse genome, the human *IGF2R* has a gICR in intron 2 (Figure 5, ICR_409 and ICR_410) [82], and according to GTEx Project data, *AIRN* is significantly expressed principally in the brain (https://gtexportal.org/home/gene/AIRN). Nevertheless, *IGF2R* is biallelically expressed in all human tissues investigated, except for sporadic monoallelic expression in the human placenta [113]. Interestingly, *AIRN* is not present in other mammals that show imprinted *IGF2R* expression, such as the dog [114] and opossum [115]. This suggests that other epigenetic modifications are involved in controlling imprinted expression at the *IGF2R* locus in these mammalian species [61]. Since the *IGF2R* is involved in carcinogenesis [116,117], human longevity [118], and cognitive ability [56], the biological function of genomic imprinting at the *IGF2R* locus in human growth, aging, and brain development needs to be more thoroughly investigated.

### Imprinting and disease susceptibility

George Orwell wrote in *Animal Farm* [119], ‘All animals are equal, but some animals are more equal than others’. The same is true of genes when it comes to disease susceptibility, and those that are ‘more equal’ are the imprinted genes. The parental allele expressed can be chosen either randomly [120] or in a parental-specific manner, as observed in genomically imprinted genes [92,121]. Genomic imprinting may be evolutionarily adaptive because of its involvement in metabolism [78] and brain development [80,122], and its potential ability to accelerate mammalian speciation [108,123]. At the same time, the presence of functionally haploid imprinted genes in the human genome can be disastrous to the health of an individual.

A number of developmental disorders in humans, such as Beckwith–Wiedemann and Silver–Russell syndromes, result not only from genomic mutations, but also from epigenetic dysregulation of imprinted genes [124]. Interestingly, Silver–Russell syndrome, a congenital disease characterized by growth retardation, is the first human disorder shown to result from epigenetically mediated imprinting defects affecting two different chromosomes [125]. Ten percent of patients present with maternal UPD of chromosome 7, while 40% to 65% show hypomethylation at the gICR upstream of *H19* at chromosome location 11p15.5 (Figure 4A), resulting in reduced IGF2 expression [124]. In contrast, hypermethylation of the *H19* gICR, with concomitant biallelic expression of *IGF2,* is associated with the overgrowth disorder, Beckwith-Wiedemann syndrome [126]. These two developmental syndromes are mirror disorders resulting from loss and gain of methylation (*i.e*. LOI) at the same ICR. LOIs at other ICRs that result in the formation of mirrored pathological conditions need to be identified.

GWAS and CNV studies have identified genomic regions linked to complex disorders such as autism, bipolar disorder, schizophrenia, and Tourette’s syndrome with a parent-of-origin inheritance preference, also indicating the involvement of imprinted genes in their etiology [127]. Two imprinted genes implicated in the development of autism and schizophrenia are 1) *DLGAP2,* a membrane-associated guanylate kinase localized at postsynaptic density in neuronal cells [109,128]; and 2) *MAGI2,* a multi-PDZ domain scaffolding protein that interacts with several different ligands in the brain [109,129,130], respectively. Thus, the role of imprinted genes in the etiology of all behavioral disorders needs to be systematically investigated, particularly in autism since its rapid increase in incidence implies the involvement of environmentally-induced alterations in the epigenome [131].

Exposure to famine conditions while *in utero* both increases the risk of developing cardiovascular disease, obesity, and diabetes [132] and also doubles the incidence of schizophrenia [133,134]. Furthermore, individuals who were prenatally exposed to famine during the 1944–1945 Dutch Hunger Winter had less DNA methylation at the *H19/IGF2* domain gICR six decades later (Figure 4A) [135]. These data suggest that a major reduction in nutrition during pregnancy – particularly during the first trimester – markedly increases psychosis formation by modifying ICRs. In contrast, the calorie-rich Western diet is predicted to potentially increase the prevalence of autism [81]. Thus, a better understanding of the role of nutrition [81] and environmental factors, like endocrine-disrupting agents [131], in the genesis of autism is needed.

Determining the role of imprinted genes in abnormal behavioral formation is challenging since brain-specific DNA methylation profiles cannot be measured in living individuals. Nevertheless, given the consistency across tissues of the parentally established epigenetic marks present in gICRs (Figure 5), they should also be detectable in peripheral tissues that are amenable to analysis. This postulate is supported by a recent study in mice where hypermethylation was observed in the ICR of *Grb10* in both the blood and liver of Pb-exposed male animals [136]. Thus, even in the absence of tissue-specific DNA methylation profiles, screening affected individuals for epigenetic disruptions in imprint regulatory elements should prove to be highly informative for ascertaining the role of genomic imprinting in environmentally-induced psychiatric conditions and other chronic diseases.

## Environmental epigenomic studies

Cathrine Hoyo established a NEST human cohort to investigate imprinting dysregulation and disease risk [137]. This is an ongoing prospective study of women and their children. It was designed to identify early exposures associated with stable epigenetic alterations in the gICRs of infants that may alter chronic disease susceptibility later in life. Between 2005 and 2011, more than 2000 pregnant women visiting prenatal clinics at Duke or Durham Regional Hospitals were enrolled. Children from these pregnancies are still being followed every two years to collect growth trajectories, disease diagnoses, and behavioral data.

These pioneering environmental epigenomic studies initially involved the measurement of gICR DNA methylation in peripheral blood lymphocytes for only a few known imprinted genes (*i.e. IGF2/H19, PLAGL1, IGF2, MEST, PEG3, MEG3, and NNAT*). They demonstrated that the methylation pattern of gICRs in children was significantly altered with maternal folic acid consumption [137], maternal and paternal obesity [138], maternal depression and antidepressant drug use [139,140], intrauterine infection [141], maternal antibiotic use [142], maternal smoking [143], and heavy metal exposure to Cd [144] and Pb [145]. Susan Murphy and her colleagues further showed that the DNA methylation of 3979 CpG sites in human sperm was associated with cannabis exposure, of which 19 were also deregulated in male tobacco smokers [146]. Of these genes, *APC2* and *RFPL2* were recently predicted to be imprinted [82]. They also showed, with an *in vitro* human spermatogenesis model, that chronic cannabis exposure altered DNA methylation of the known paternally expressed imprinted genes *SGCE*, *GRB10*, and *PEG3,* and a candidate imprinted autism gene, *HCN1* [82,147].

## The human imprintome

Nevertheless, to enhance our understanding of human behavior and disease formation, it was important to define both the complete repertoire of human imprinted genes and their regulatory elements - the human imprintome [90,148]. Following the publication of the human genome in 2001 [149,150], we first used computer machine learning algorithms or AI approaches to predict the genome-wide imprint status of human genes from sequence features [109]. Of the 102 annotated genes identified to be potentially imprinted, we have now identified ICRs for 35 of them (34%) (Supplementary Table S4, [83[82]), providing additional evidence that they are indeed imprinted. Two of these computationally identified putative imprinted genes, *DLGAP2* and *KCNK9,* were also demonstrated experimentally to be paternally and maternally expressed, respectively. *DLGAP2* is a membrane-associated protein that plays a role in synapse organization and signaling in neuronal cells, and as previously stated, is implicated in the etiology of autism [128]. Since it is imprinted, it can potentially be altered both genetically and/or epigenetically in the pathogenesis of autism. Maternally expressed *KCNK9* encodes for the pH-sensitive potassium channel protein, TASK3. TASK3 is present at the plasma membrane and regulates membrane depolarization in response to acidosis via inhibition of the background potassium current. Maternal germline inactivation of the maternally expressed allele of *KCNK9* results in Birk–Barel syndrome [151], and *KCNK9* loss of imprinting is also prevalent in triple-negative breast cancer [152].

We recently determined the human imprintome by performing WGBS of DNA derived from tissues arising from the three germ layers (i.e., brain, liver, and kidney) and from the egg and sperm [82]. The gICRs in somatic cells will be 50% methylated because the gametes are either methylated or unmethylated at the gICR locations (Figure 5). We identified 1,488 hemi-methylated candidate ICRs. Gamete methylation approached 0% or 100% in 332 gICRs (i.e., 178 paternally and 154 maternally methylated), supporting parent-of-origin-specific methylation. This first draft of the human imprintome (https://humanicr.org/) allows for a more systematic determination of the importance of imprinting dysregulation in the formation of human diseases and behavioral disorders.

We recently used the human imprintome coupled with WGBS to determine whether aberrant DNA methylation at ICRs is associated with Alzheimer's disease (AD) [153]. This study showed that 120 candidate ICRs varied significantly between AD cases and controls. The number of ICRs with altered methylation in individuals with AD is three times higher in non-Hispanic blacks (NHBs) than in non-Hispanic whites (NHWs), suggesting a possible reason for the higher prevalence of AD in NHBs than in NHWs [154]. Interestingly, only two ICRs are common to both NHBs and NHWs, and they are proximal to the inflammasome gene, *NLRP1*, and a known imprinted gene, *MEST/MESTIT1*. These findings indicate, for the first time, that early developmental alterations in DNA methylation of regions regulating genomic imprinting may contribute to AD risk, and that this epigenetic risk differs between NHBs and NHWs.

**Figure 6:**
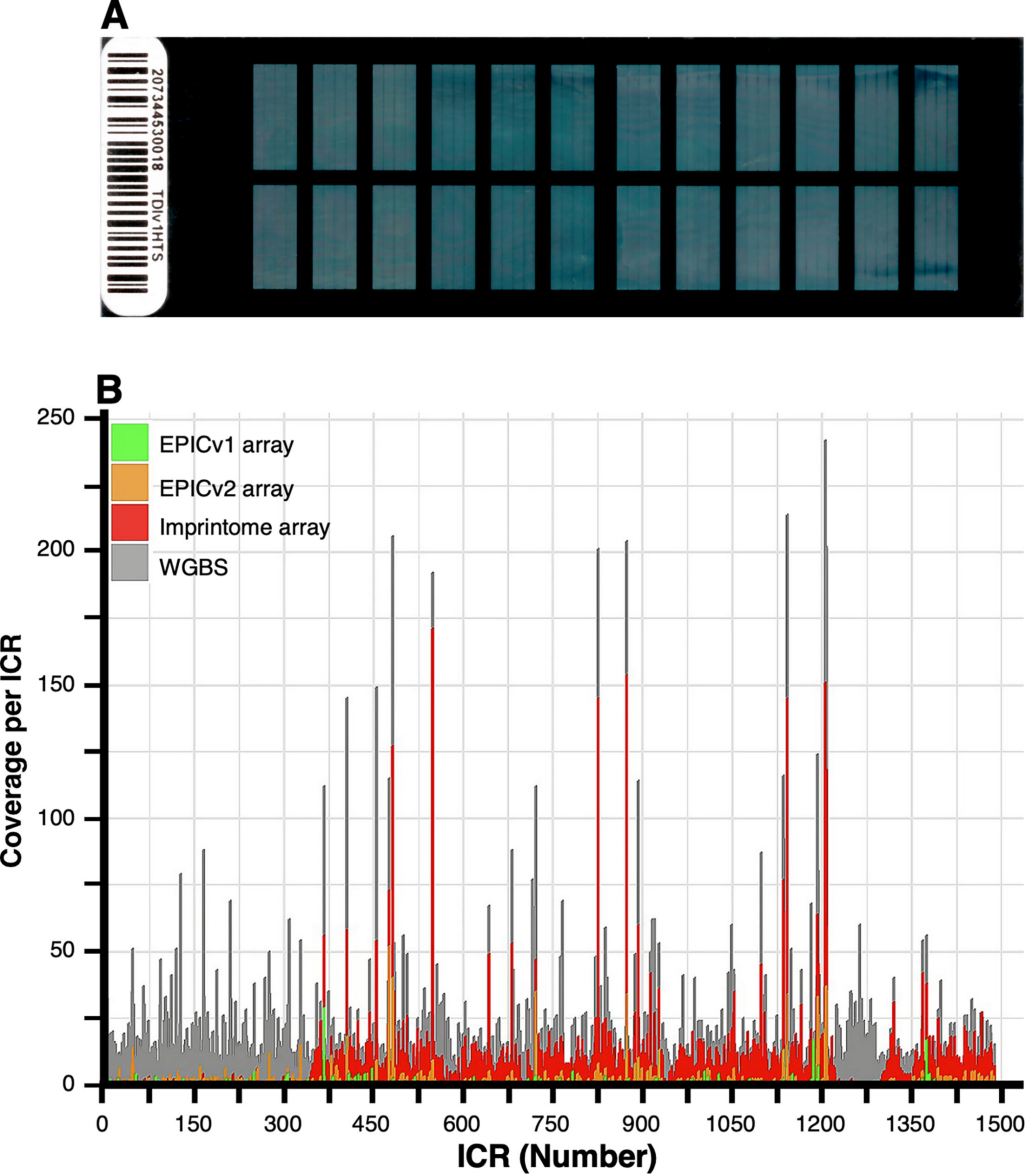
Infinium DNA methylation array for the human imprintome. (**A**) *Human imprintome array* for the DNA methylation ICR analysis of 24 samples. (**B**) Coverage of the 1488 ICRs in the human imprintome is shown for WGBS (100% ICR coverage), the human imprintome array (73% ICR coverage), EPICv1 array (10% ICR coverage) (Illumina, Inc. San Diego, CA), and EPICv2 array ((36% ICR coverage) (Illumina, Inc. San Diego, CA) [82,83]). ICR, imprint control region.

WGBS is the most precise method to interrogate gICRs for DNA methylation changes in environmental epigenomic studies; however, it is expensive, requires high coverage, and it is computationally intensive. To address this deficiency, a custom methylation array containing 22,819 probes was developed in collaboration with TruDiagnostics (Lexington, KY) (Figure 6A) [83]. It contains 9,757 probes mapping to 1,088 out of the 1,488 candidate ICRs in the human imprintome (Figure 6B).

This custom array should help accelerate the rapid screening for gICRs associated with a wide range of chronic diseases and exposures, thereby advancing our understanding of genomic imprinting and its relevance in development and disease formation throughout the life course.

## Conclusion

Traditional research to determine the role of gene-environment interactions in disease risk examines the relationship among disease susceptibility, environmental exposures, and germline mutations. Such research efforts have highlighted the importance of genotype in human diseases; however, it has become clear that full understanding of the mechanisms by which disease risk is altered by environmental exposures will require epigenetic mechanisms to also be taken into account.

**Summary**

- Two epigenetically regulated subsets of genes that link environmental exposures early in development to adult diseases are imprinted genes and those with metastable epialleles. The A^vy^ mouse metastable epiallele demonstrated, for the first time, that early developmental exposures to nutritional (i.e., methyl donors and genistein), chemical (i.e., BPA), and physical agents (i.e., LDIR) alter disease susceptibility in adulthood by modifying DNA methylation. Thus, human health and disease stem not only from genetic mutations but also from changes in the epigenome.
- Genomic imprinting evolved about 150 million years ago in a common ancestor to Therians, resulting in the formation of disease susceptibility loci since only a single mutation or epigenetic event is needed to cause loss of gene function. Consequently, deregulation of imprinted gene expression is involved in a variety of chronic human diseases and behavioral disorders.
- The level of methylation at gICRs in the human imprintome [82] and metastable epialleles (i.e. CoRSIVs) [24] is independent of tissue type, making them ideal for DOHaD epidemiology studies where the tissue of interest cannot be obtained. The human imprintome and metastable epiallele control regions in humans have been identified [24,82]. Although a human imprintome array is available to facilitate the determination of gICRs deregulated in chronic diseases and behavioral disorders when exposed to environmental factors [83], a similar array for CoRSIVs is presently unavailable [155]. This deficiency needs to be corrected in order to more systematically determine the role of these two subsets of epigenetically regulated genes in human health and disease and risk to LDIR.

## Abbreviations

AD: Alzheimer's disease
AI: artificial intelligence
A^vy^: Agouti viable yellow
BPA: Bisphenol A
CT: computed tomography
CTCF: CCCTC-binding factor
CTCFL: CCCTC-binding factor like
CoRSIV: correlated regions of systemic interindividual variation
DEN: diethylnitrosamine
DNMT1: DNA methyltransferase 1
DNMT3A: DNA methyltransferase 3 alpha
DNMT3B: DNA methyltransferase 3 beta
DOHaD: Developmental origins of health and disease
DPPA3: Developmental pluripotency-associated 3
EMB: embryo
GRB10: growth factor receptor bound protein 10
HCN1: hyperpolarization activated cyclic nucleotide gated potassium channel 1
HDACs: histone deacetylases
IAP: intracisternal A-particle
ICR: imprint control region
LDIR: Low dose ionizing radiation
MC4R: Melanocortin 4 receptor
MYA: million years ago
NHBs: non-Hispanic blacks
NHWs: non-Hispanic whites
PEG3: paternally expressed gene 3
PGCs: primordial germ cells
PS1A: Pseudoexon 1A
SGCE: Sarcoglycan epsilon
TASK3: Tandem pore domain acid-sensitive K+ channel 3
TB: Trophoblasts
TNBC: Triple negative breast cancer
Tme: T-maternal effect
WGBS: whole genome bisulfite sequencing
WT: wildtype
YS: Yolk sac
gICR: Germline imprint control region
sICR: somatic imprint control region
